# Factors associated with satisfaction of the australian rural resident medical officer cadetship program: results from a cross-sectional study

**DOI:** 10.1186/s12909-024-05737-z

**Published:** 2024-07-29

**Authors:** Phillipa Kensit, Md Irteja Islam, Robyn Ramsden, Louise Geddes, Yann Guisard, Chris Russell, Alexandra Martiniuk

**Affiliations:** 1 The University of Notre Dame Australia, Lithgow Clinical School, Col Drewe Drive, Lithgow, NSW Australia; 2 Rural Doctors Network, 7/33 Chandos, St. Leonards, NSW Australia; 3 Sydney School of Public Health, Faculty of Medicine and Health, The University of Sydney, Edward Ford Building, A27 Fisher Road, Camperdown Sydney, NSW 2006 Australia; 4 Centre for Health Research and Faculty of Health, Engineering and Sciences, The University of Southern Queensland, West Street, Darling Heights, Toowoomba, QLD 4350 Australia; 5 Office of the Chief Scientist, The George Institute for Global Health, Level 5/1 King Street, Newtown Sydney, NSW 2042 Australia; 6 Dalla Lana School of Public Health, The University of Toronto, 155 College St Room 500, Toronto, ON M5T 3M7 Canada; 7 Charles Sturt University, School of Rural Medicine, Faculty of Science and Health, Orange Campus, 346 Leeds Parade, Orange, NSW 2800 Australia; 8 Deakin University School of Health and Social Development, 221 Burwood Highway, Burwood, VIC Australia

**Keywords:** Rural, Rural medical workforce, Medical student, Scholarship programs, Financial support, Networking, Rural resident, Medical officer cadetship program, Cadetship, Return of service

## Abstract

**Background:**

Australian Rural Resident Medical Officer Cadetships are awarded to medical students interested in a rural medical career. The Rural Residential Medical Officer Cadetship Program (Cadetship Program) is administered by the Rural Doctors Network on behalf of the NSW Ministry of Health. This study aimed to assess the overall experience of medical students and key factors that contributed to their satisfaction with the Cadetship Program.

**Methods:**

A quantitative cross-sectional study was conducted among 107 former cadets who had completed the Cadetship Program. Data on medical students’ experience with the Cadetship Program (outcome variable) and potential explanatory variables were collected using a structured self-administered questionnaire. Explanatory variables included gender, geographical location, rural health club membership, rural clinical school attendance, financial support, mentorship benefits, networking opportunities, influence on career decisions, opportunity for preferential placements, and relocation. Both bivariate (Pearson’s chi-squared test) and multiple logistic regression analysis were employed to identify the factors associated with medical students’ overall experience with the Cadetship Program. The non-linear analysis was weighted to represent the rural/remote health workforce, in Stata/SE 14.1.

**Results:**

Our results indicate that 91% of medical students were satisfied with the Cadetship Program. The logistic regression model identified two significant predictors of a positive experience with the Cadetship Program. Medical students who perceived financial support as beneficial were significantly more likely to report a satisfactory program experience (aOR = 6.22, 95% CI: 1.36–28.44, *p* = 0.019) than those who perceived financial support as not beneficial. Similarly, those who valued networking opportunities were more likely to have a positive view of their cadetship experience (aOR = 10.06, 95% CI: 1.11–91.06, *p* = 0.040) than their counterparts.

**Conclusion:**

Our study found that students who valued financial support and networking opportunities had the most positive views of the Cadetship Program. These findings demonstrate that the Cadetship Program may be most helpful for those who need financial support and for students who seek networking opportunities. These findings increase our knowledge about the characteristics of medical students who have the most positive experiences with the Cadetship Program. They help us to understand the mechanisms of influence of such programs on individuals’ decisions to be part of the future rural health workforce.

**Supplementary Information:**

The online version contains supplementary material available at 10.1186/s12909-024-05737-z.

## Introduction

According to the Australian Institute of Health and Welfare (AIHW) (2020), approximately 7 million people – 28% of the Australian population, live in rural Australia [1]. People residing outside of metropolitan centres experience poorer health outcomes with higher rates of hospitalisations, early deaths, and injury, due to multiple factors with one being decreased access to, and utilisation of, health services and providers [1]. Access to, and utilisation of, healthcare services and providers in regional, rural and remote areas is reliant on the availability of a sustainable rural health workforce [2–4]. Although healthcare is the largest industry in regional New South Wales (NSW) (14.6% of the regional workforce), the distribution of doctors and other key health professionals contrasts greatly with that of metropolitan areas [5]. AIHW (2023) data indicate that in 2021 there were 74.6 General Practitioners and 9.8 specialists per 100,000 population in small rural towns compared to 107.0 General Practitioners and 152.3 specialists per 100,000 population in metropolitan Australia. The challenges associated with attracting, recruiting, and retaining a rural health workforce contribute to poorer health outcomes for people living in rural communities [6, 7]. Similar challenges are observed globally. For instance, health workforce recruitment and retention issues amongst rural physicians were found in recent systematic reviews in Canada, the USA and Europe [8–10].

Comprehensive rural health workforce solutions to help address ongoing health workforce shortages in rural and remote settings are needed [2, 11]. One successful strategy for recruiting health professionals to rural areas was found to be training in rural areas regardless of the background of the health professional (rural or metropolitan) [3, 12, 13]. Despite this, only 13% of specialist medical training occurs in such locations [14]. Although rural origin remains a critical factor influencing medical practitioners’ decision to train and practice rurally, programs that include students from all geographic locations are necessary to address current workforce issues [15, 16]. While a recent systematic review showed a discrepancy in the ideal rural clinical placement length, most studies reported that an organised, well-funded, rural placement or rural clinical school program was positively associated with increased rural intentions and graduate rural employment [17, 18].

Several studies have explored the impact of financial incentives, including scholarships and return of service (ROS) components, on the recruitment and retention of healthcare workers in rural areas [4, 19, 20]. Research has indicated varying degrees of success with these approaches, highlighting the importance of additional factors such as the design of the incentive programs, the characteristics of the target population, and the support structures in place for healthcare workers in rural settings [4, 19, 21]. Other studies found that the ‘return of service’ components of a scholarship program have the potential to increase attraction and recruitment of health professionals to regional, rural and remote locations in the short term, but are less effective for longer-term retention of workers [22, 23]. Looking at a different approach, Wyatt et al., (2018) reported that the ‘Towards Rural and Outback Health Professionals in Queensland’ rural health club strengthened medical students’ interest in a rural career [24].

Another successful strategy has involved the early activation and development of health career aspirations and intentions among young people in these settings [25]. Talent management refers to the strategic process of attracting, identifying, developing, and retaining skilled and capable individuals to maximise their potential and performance [26]. Several studies have shown the association between talent management in healthcare and staff effectiveness and quality of services [26–29], attraction and retention of health professionals [26, 30–32], staff development [26, 28, 33] and work climate, work satisfaction, culture and leadership [26, 29]. A study conducted by Devine et al., (2013) of the ‘Queensland Health Rural Scholarship Scheme (Allied Health)’ identified that regional placements before and during rural tenure were a means of developing both skills and networks [19]. A study by Cabral et al., (2019) on nursing leadership identified that coaching, mentoring, and support networks were crucial to developing and maintaining nursing leadership talent [28]. Furthermore, this study found that talent networks reduce professional isolation [28]. Professional and clinical support and supervision, a supportive work environment and culture, mentoring and professional development were also found to be important factors for retention [19].

The Rural Resident Medical Officer Cadetship Program (Cadetship Program) established in 1989 provides scholarship opportunities to incentivise rural health and practice during medical students’ studies [16, 34]. The Cadetship Program is administered by the Rural Doctors Network on behalf of the Rural Doctors Network. The program provides financial support to medical students interested in undertaking a medical career in rural NSW with a return-of-service (ROS) component. In addition, the program provides a structured talent management component that involves mentorship, networking, professional development, and rural immersion experiences. Despite the established role of scholarship programs in supporting rural healthcare careers, there is little research on the additional benefit of the talent management component of a scholarship program for attracting and retaining health professionals in rural careers [19].

Thus, this study aimed to identify the elements of the Cadetship Program which medical students found beneficial. The authors hypothesise that financial support, networking opportunities, and mentorship provided by the Cadetship Program will positively influence participants’ overall experience and contribute to their long-term career decisions in favour of rural health practice. This paper reports on the overall experience and the important factors associated with satisfaction with the Cadetship Program among respondents who completed the program and are working as medical officers.

## Methods

### Study design

This study was conducted among the recipients of the Cadetship Program following a retrospective cross-sectional study design.

The cadetship program description:

The Cadetship Program established in 1989, provides successful applicants up to $15,000 per year for the final two years of their medical degree or Indigenous students $30,000 spread throughout their study. In return, students agree to undertake two of the first three years of their hospital training in an eligible rural NSW hospital. In their final year of study, cadets apply for an intern position through the Rural Preferential Recruitment (RPR) process or Aboriginal Medical Workforce pathway administered by the Health, Education and Training Institute (HETI). Rural service must be undertaken in an eligible regional NSW hospital. Eligible hospitals are located in Tamworth, Dubbo, Orange, Wagga Wagga and Albury, NSW.

In addition to receiving financial support and the need to complete their ROS in a major regional hospital, the Cadetship also provides a structured talent management program. In this program talent management comprises various practices aimed at maximising the capability of medical students and Junior Medical Officers to equip them with appropriate motivation, skills and networks to work in rural and remote communities. A major component of the Rural Doctors Network talent management approach is to support medical students to align their skills and capabilities with the needs of rural and remote communities, health workforce practice and clinician wellbeing. The program provides a range of activities and events to support students beyond their clinical training. These include attendance at conferences and cadet weekends, personalised support, networking, mentorship, and learning opportunities.

### Study setting and data collection

In October 2022, the SurveyMonkey® survey was purposively distributed via email to all 373 recipients of the Cadetship Program from 1989—2021 for voluntary completion. We included recipients over this time to maximise the numbers completing the survey to determine satisfaction and rural retention outcomes. However, only 114 agreed to participate – out of which 107 completed the self-administered survey questionnaire with written informed consent (28.7%). No incentive was offered to the study participants for participation and all respondents were assured of complete confidentiality and anonymity.

### Measures

Based on the results of previous research conducted by the Rural Doctors Network a structured online questionnaire was designed to collect information on sociodemographic variables and information related to the Cadetship Program. The sociodemographic variables include gender (male, female); geographical location (metropolitan, rural/remote); Member of rural health club (no, yes); attended rural clinical school (no, yes); role of financial support (not beneficial, beneficial); mentorship benefits (no, yes); networking opportunities (no, yes); influence on career decisions (no, yes), preferential placements opportunity (agree, disagree), and effect of relocation from metropolitan to rural and vice versa (no, yes).

While questions assessing attitudes towards the Cadetship Program included the overall experience, and whether the participants would recommend the program to other medical students, in this study, participants’ overall experience with the Cadetship Program was selected as the main outcome variable. Study participants’ overall experience was measured with the following question: ‘Overall, how would you rate your overall experience of the cadetship program?’, rated on a five-point Likert scale (very poor, poor, fair, good, excellent). For the descriptive analyses, we created a dichotomised variable, ‘Participants’ cadetship experience’, from the responses. Participants who responded ‘good’ or ‘excellent’ were classified as ‘satisfied’ (coded as 1), while those who answered, ‘very poor’, ‘poor’, or ‘fair’ were classified as ‘dissatisfied’ (coded as 0). The dichotomisation facilitated the identification of trends and associations which allowed for clearer data analysis and interpretation. Additionally, using this format facilitated a more direct comparison and clearer conclusions about the factors influencing satisfaction.

### Statistical analysis

At first, the characteristics of the sample (*n* = 107) were described using descriptive statistics. This included the frequency (n) and percentages (%) along with a 95% confidence interval (CI) for all possible sociodemographic variables. Then, we used a Pearson’s correlation coefficient matrix to measure the linear correlation between selected variables. Following this, bivariate analyses using Pearson’s Chi-squared tests were performed to investigate the distributions of explanatory variables over the main outcome variable, which was the overall experience of medical students with the Cadetship Program. Subsequently, multiple logistic regression models were utilised to determine the factors linked to the overall experience of the Cadetship Program. Only the statistically significant factors (*p* < 0.05) in bivariate analysis were included in the adjusted logistic model. The logistic regression analysis yielded adjusted odds ratios (aOR) together with their matching 95% confidence intervals (CI) and p-values. Data were processed using Stata/SE 14.1 (Stata Corp, College Station, TX, USA) and *p* < 0.05 was considered statistically significant.

Furthermore, the assumptions of the regression model were evaluated. For instance, McFadden’s R^2^ for model performance and variance inflation factor (VIF) statistics were assessed for multicollinearity among the predictor variables in the model. All the estimates were weighted to represent the rural/remote health workforce in the Australian population, and the ‘SVY’ command of Stata/SE 14.1 was used to account for survey design and to adjust the results according to the survey setting.

### Ethics

The study was ethically approved by the North Coast NSW Human Research Ethics Committee [Ref. No. 2020/ETH03117]. Moreover, following the guidelines outlined in the National Statement on Ethical Conduct in Human Research, this research used a routinely collected anonymous dataset. The results obtained from this dataset are also presented in an unidentifiable form, which fulfils the University of Sydney Research Ethics Board’s Outcome A and does not require additional ethical committee approval from the University.

## Results

The sample included 107 cadets who were the recipients of the Cadetship Program and completed the survey questionnaire distributed by the Rural Doctors Network. Table 1 summarises the characteristics of the survey respondents. Most respondents were females (*n* = 65, 60.7%), more than half of the participants (*n* = 60, 56%) were from rural/remote areas and similar percentages were rural health club members (*n* = 60, 56%). Nearly 89% (*n* = 95) of the sample reported the financial support as beneficial (*n* = 95, 88.8%), around 38% (*n* = 41) benefited from having a mentor, and more than 50% (*n* = 55) agreed to the networking opportunity during the Cadetship Program. Approximately 75% (*n* = 80) of the participants reported that the Cadetship Program influenced career decisions, 42% (*n* = 45) had the opportunity for rural preferential recruitment, and the majority of the participants (*n* = 95, 88.8%) reported there were no effect of relocation during the program.

**Table 1:** Sample (*n* = 107) characteristics of the population who completed the Cadetship Program survey in 2022

Characteristics	n (%)
Gender	
Male	42 (39.3)
Female	65 (60.7)
Area of current residence	
Metropolitan	47 (44.0)
Rural/Remote	60 (56.0)
Rural health club membership^1^	
No	47 (44.0)
Yes	60 (56.0)
Role of financial support^2^	
Not Beneficial	12 (11.2)
Beneficial	95 (88.8)
Benefit of having a mentor^3^	
No	66 (61.7)
Yes	41 (38.3)
Networking opportunity ^4^	
No	52 (48.6)
Yes	55 (51.4)
Influence on career decisions^5^	
No	27 (25.2)
Yes	80 (74.8)
Preferential placement opportunity^6^	
Disagree	62 (58.0)
Agree	45 (42.1)
Effect of relocation^7^	
No	95 (88.8)
Yes	12 (11.2)

Notes:

^1^ Whether the participant is a member of rural health club

^2^ Whether financial supports influenced participants to apply for the Cadetship

^3^ Whether the opportunity to receive mentoring support from Rural Doctors Network during the Cadetship influenced the decision to apply

^4^ Whether the opportunity to access networking opportunities during their Cadetship influenced the decision to apply

^5^Whether time spent in a Regional Hospital influenced their career decision

^6^ Whether bettering Rural Preferential Recruitment (RPR) opportunities because an applicant is a Cadet influenced the decision to apply

^7^ Whether relocating to a regional area to complete the Return of Service influenced the decision to apply

**Fig. 1:**
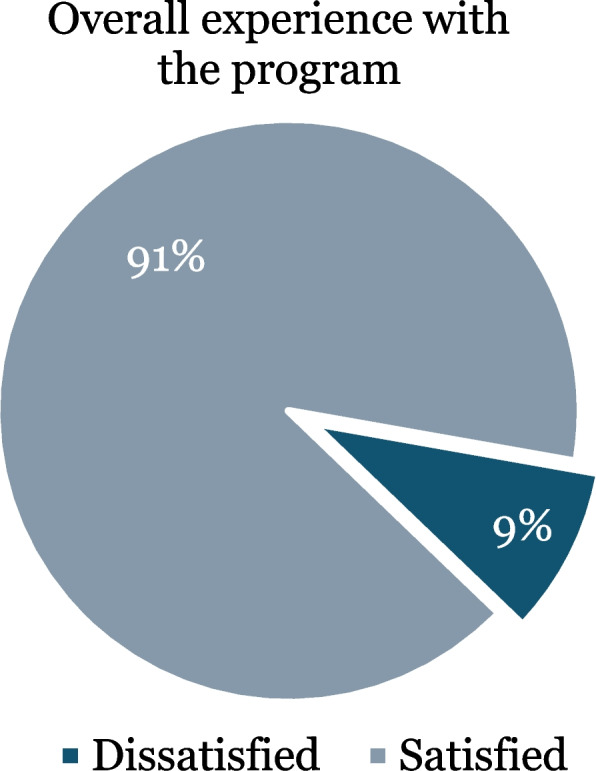
Study participants’ experience (Satisfied vs Dissatisfied) with the Cadetship Program

The rates of overall experience of study participants with the Cadetship Program are depicted in Fig. 1. Out of a total of 107 participants, the majority (99, 91%) were satisfied with the scholarship program.

Table 2 illustrates the correlation coefficient matrix among the selected study variables. These data suggest correlations between the variables used as potential predictors for our regression model, however only financial support and networking opportunities are found to be significantly positively correlated with the participants overall experience with the Cadetship Program.

**Table 2:** Correlation matrix between selected variables

	1. Gender	2. Area of residence	3. Rural health club membership	4. Role of financial support	5. Benefit of having mentors	6. Networking opportunity	7. Influence on career decisions	8. Preferential placement opportunity	9. Effect of Relocation	10. Experience with [Name of program]
1. Gender	1									
2. Area of residence	-0.210*	1								
3. Rural health club membership	-0.094	0.089	1							
4. Role of financial support	0.018	0.044	-0.016	1						
5. Benefit of having mentors	0.122	0.078	0.155	0.097	1					
6. Networking opportunity	-0.016	0.194*	0.382*	0.129	0.574*	1				
7. Influence on career decisions	0.062	0.093	0.18	0.202*	0.236*	0.253*	1			
8. Preferential placement opportunity	-0.013	-0.009	0.258*	0.302*	0.263*	0.260*	0.277*	1		
9. Effect of Relocation	-0.078	0.136	0.016	0.033	-0.036	-0.01	0.206*	-0.063	1	
10. Experience with [Name of program]	0.071	-0.025	0.104	0.292*	0.187	0.266*	0.109	0.144	-0.089	1

The results from the bivariate analysis between explanatory variables and participants’ overall experience with the Cadetship Program are in Table 3. Variables such as financial support, the benefit of having mentors and networking opportunities during the Cadetship Program were significantly associated with the participants' overall experience with the Cadetship Program (*p* < 0.05 for all).

**Table 3:** Bivariate association between sociodemographic variables and Participants' experience with the Cadetship Program

	Dissatisfied	Satisfied	Pearson χ^2^ (p-value)
	n (%)	n (%)	
Gender			0.534 (0.46)
Male	5 (50.0)	37 (38.1)	
Female	5 (50.0)	60 (61.9)	
Area of residence			0.069 (0.79)
Metropolitan	4 (40.0)	43 (44.3)	
Rural/Remote	6 (60.0)	54 (55.7)	
Rural health club membership			1.157 (0.28)
No	6 (60.0)	41 (42.3)	
Yes	4 (40.0)	56 (57.7)	
Role of financial support			9.179 (0.000***)
Not Beneficial	4 (40.0)	8 (8.3)	
Beneficial	6 (60.0)	89 (91.8)	
Benefit of having mentors			3.743 (0.05*)
No	9 (90.0)	57 (58.8)	
Yes	1 (10.0)	40 (41.2)	
Networking opportunity			7.5693 (0.01**)
No	9 (90.0)	43 (44.3)	
Yes	1 (10.0)	54 (55.7)	
Influence on career decisions			1.275 (0.26)
No	4 (40.0)	23 (23.7)	
Yes	6 (60.0)	74 (76.3)	
Preferential placement opportunity			2.202 (0.134)
Disagree	8 (80.0)	54 (55.7)	
Agree	2 (20.0)	43 (44.3)	
Effect of relocation			0.855 (0.36)
No	8 (80.0)	87 (89.7)	
Yes	2 (20.0)	10 (10.3)	

Level of significance: *p < 0.05, **p < 0.01, ***p < 0.001

Table 4 describes the results from the binary logistic regression models that identified the predictors of study participants’ overall experience with the Cadetship Program. Statistically significant variables (*p* < 0.05) from the unadjusted model were included in the adjusted model. In the adjusted model, respondents who reported financial support as beneficial were 6.22 times (95% CI: 1.36–28.44, *p* = 0.019) more likely to be satisfied with the Cadetship Program than those who reported financial support as not beneficial. Further, those who reported that the Cadetship Program provided network opportunities were 10.06 times (95% CI: 1.11–91.07, *p* = 0.040) more likely to be satisfied with the Cadetship Program than their counterparts.

**Table 4:** Determinants of participant's overall experience with the Cadetship Program—Binary logistic model

	Experience with the [Name of program]		
	COR (95% CI)	AOR (95% CI)	VIF
Gender (ref. Male)			
Female	1.62 (0.43, 610)	-	
Area of residence (ref. Metropolitan)			
Rural/Remote	0.83 (0.21, 3.22)	-	
Rural health club membership (ref. No)			
Yes	2.04 (0.53, 7.89)	-	
Role of financial support (ref. Not beneficial)			1.02
Beneficial	7.41** (1.68, 32.61)	6.22* (1.36, 28.44)	
Benefit of having mentors (ref. No)			
Yes	6.31 (0.74, 53.61)	-	
Networking opportunity (ref. No)			1.02
Yes	11.30* (1.33, 95.88)	10.06* (1.11, 91.07)	
Influence on career decisions (ref. No)			
Yes	2.14 (0.54, 8.44)	-	
Preferential placement opportunity (ref. Disagree)			
Agree	3.18 (0.62, 16.19)	-	
Effect of relocation (ref. No)			
Yes	0.45 (0.08, 2.53)	-	
Model statistics			
McKelvey and Zavoina's R^2^	-	0.360	
Mean VIF	-	1.02	

*COR *Crude Odds Ratio, *AOR *Adjusted Odds Ratio, *CI *Confidence Interval, *VIF *Variance Inflation Factor

Level of significance: ***p < 0.001, **p < 0.01, *p < 0.05

Variables found significant in unadjusted models were included in the adjusted model

Furthermore, Table 4 reveals that the VIF test, with a mean value of 1.02, validated the lack of multicollinearity in the adjusted model, and McFadden’s R^2^ value of 0.360 indicated that the model was well-fitted.

## Discussion

This study investigated the medical students’ overall experience with the Cadetship Program and determinants of overall experience were identified. The current study revealed that more than 90% of respondents were satisfied with the Cadetship Program. Moreover, this study also found that financial support and networking possibilities were important elements among the predictors examined in our study that predicted overall satisfaction with the Cadetship Program. Although the other variables did not show statistical significance in the bivariate logistic models, such as area of residence for example, we strongly suggest that they should be further explored in future models as they are often reported as predictors for rural practice or intention to practice rurally. We now explore these variables in further detail, and we suggest that they form facets of a desirable trait that we tentatively name “prevocational trainee capability”.

### Financial support

Financial support was the most critical variable in enhancing Cadetship satisfaction (Table 4). The overwhelming majority of respondents (*n* = 95, 88.8%) highlighted financial support as the pivotal variable in their satisfaction with the Cadetship (Table 4). Relatedly, broader research has found utility in financial incentives for promoting rural practice [19, 20]. However, while financial support is important to cadets, a study by Schofield et al., (2019) found that despite being more inclined to choose rural placements when financially supported, students frequently encountered additional economic barriers that deter them from these opportunities [35]. These include lost income and increased living expenses affecting students relocating to rural areas for their placements [35]. Swami and Scott (2021) in their examination of the General Practice Rural Incentives Program in Australia found that while financial incentives increased the number of GPs in certain areas, they alone may not be sufficient to address rural medical workforce shortages, suggesting the significance of incentive program design and support systems [4].

### Networking

Networking opportunities are associated with satisfaction with the Cadetship Program. Networking opportunities alone were the second strongest predictor of satisfaction (*p*-value 0.01). Examples of networking opportunities include sponsored attendance at several rural conferences including the Rural GP Conference in Sydney, the Rural GPs Refresher Conference in Port Macquarie and regionally located cadet weekends among others. Networking opportunities were most significantly related to the benefit of having mentors (Table 2). Cadetship networking engagements are structured events with professional and social activities for students and practising rural health professionals. The role of networking in talent management aligns with previous literature [26, 28, 36]. On an interpersonal level, evidence suggests that collegial networks may support practitioners in developing meaningful relationships, leading to a greater commitment and increased staff retention (36), as well as providing shared knowledge, friendship and support [37] and job satisfaction [38, 39]. Recent literature also suggests that networks provide resilience and capability for rural doctors to cope with stress [40–42]. For example, Couper et al., (2022) in a study of rural physicians from different countries during the COVID-19 pandemic found clinical courage and resilience gained through teamwork-supported emotional coping [40]. Likewise, Walters et al., (2021) in their study on clinical courage and capability found that medical professional relationships including those with each other, with the local members of their healthcare team, and with other colleagues outside their immediate community of practice support them to continue facing their clinical responsibilities in rural areas [42]. In a study by Burgis-Karthala et al., (2024), the authors highlighted the importance of doctors developing connectedness across geographic, personal, and professional domains. Importantly helping medical students participating in the Cadetship Program to develop supportive interrelationships through networking may help to maximise retention in rural practice [43].

### Mentorship

The findings from the survey also highlighted the importance of mentorship to participants' satisfaction with the Cadetship. Having a mentor was significantly found to be associated with medical students' satisfaction with the Cadetship in bivariate analysis (Table 2). Although definitions of mentoring vary, mentoring relates to two main things; psychosocial support and an organically formed relationship between mentor/mentee, and professional guidance through career support including skill development and professional advice [44, 45]. For example, Devine et al., (2013) and Hill-Jarrett et al., (2023) indicate positive mentor–mentee relationships positively impact future career decisions further supporting its role in the Cadetship [19, 46]. The close conceptual relationship between networking and mentorship suggests that structured networking activities such as those provided through the Cadetship are important for cadets. This is consistent with the literature and reflects the intrinsic relationship between networking opportunities and mentoring [19].

### Gender

Gender was not related to the satisfaction of the Cadetship in the 2022 survey. The absence of statistical significance in differences between genders shows that both men and women are equally likely to engage with the Cadetship and report satisfaction with the financial and networking aspects of the program. Although research is limited regarding rural medical scholarship program satisfaction and gender, another paper by Aljerian (2022) exploring variables associated with medical residents’ satisfaction levels also illustrates that gender did not affect satisfaction scores among participants [47]. These findings are also consistent with the work of Playford et al. (2020) who also found no gender differences in future career decisions and rural practice despite having fewer females in their final cohort [48].

While not a primary focus of this study, the Rural Preferential Recruitment (RPR) pathway may be an important factor in the recruitment and retention of a rural health workforce, including GP and non-GP specialists. RPR is a strategy that typically involves giving preference to candidates who are willing to work in rural and underserved areas during the recruitment process for medical and other health-related positions [49, 50]. It is a merit-based recruitment process for final-year medical students who are interested in working in a rural setting, including eligible sites for return of services [51]. McGrail et al., (2018) suggest that policies that prioritise the selection of prospective medical students from rural areas with medical workforce shortages could lead to improved outcomes for the rural medical workforce [52]. Opportunities relating to the rural preference recruitment process did not critically influence Cadetship satisfaction. RPR did however correlate significantly with every other proposed predictor variable except for genderAlthough RPR remains the dominant recruitment pathway, in recent years, rural hospitals have had trouble filling their intern places and have needed to draw from additional pathways [49]. Although the Cadetship does not guarantee first preference for Rural Preferential Recruitment, it is suggested it is helpful in the process. The reasons behind this are not explored in the paper but anecdotally relate to demonstrated rural practice intent and Cadetship experiences such as Conference attendance, rural placement experience and performed relationships with regional hospital staff through networking activities. Although RPR was not a variable highly associated with Cadetship satisfaction, there is a significant relationship with other explanatory variables of the program with significance (Tables 1, 2 and 4). For example, those who valued RPR opportunities also answered favourably regarding rural health club membership, the role of financial support, the benefit of having mentors, networking opportunities and the positive influence of career decisions. Although these data are not found to be predictors of program satisfaction, they may still suggest that RPR is “front of mind” for cadets. One may also suggest that given the rural ROS obligations associated with the Cadetship, cadets are more focused on the skills required to practice in a rural setting, than the opportunity to practice rurally per se.

While the findings of this study provide important insights into the variables influencing the satisfaction of medical students with the Cadetship, it also embodies several limitations. Firstly, the study was limited to 107 medical students, which may not capture the full diversity of experiences and perspectives within the program. Secondly, there is potential for selection bias, as participation in the survey was voluntary, and thus the responses may not fully represent the entire population of program participants. The design of this study is appropriate to explore the association between variables but does not infer causality between the program elements and student satisfaction. Finally, our study reports on one point in time. A longitudinal study could provide insights into how perceptions of the program evolve and the impact on career trajectories. Further exploration into the talent management aspects of the program would be beneficial to determine how these impact on the satisfaction, effectiveness, and sustainability of the Cadetship. Participants highly valued the networking and mentorship aspects of the program, and it would be useful to further explore the satisfaction levels and relationship between the various aspects of the talent management approach.

## Conclusion

This study increases knowledge regarding the factors that enhance participants’ satisfaction with the Cadetship Program in NSW, Australia. In line with our hypothesis, the findings highlight the significance of financial support and networking opportunities as primary drivers of a positive experience within the program. Notably, these have the potential to influence long-term career decisions towards rural health practice and should therefore remain a strong feature of the Cadetship Program. The absence of significant differences in satisfaction related to gender or rural versus metropolitan background suggests that the program’s appeal is broadly applicable, supporting a diverse group of medical students. Furthermore, the study highlights the role of mentorship and the integration of rural health club memberships as complementary supports that enrich the cadet experience. We suggest here that these variables are desirable facets of the capability of prevocational trainees. Moving forward, it is imperative that the Cadetship, and similar programs, continue to adapt and evolve based on feedback and investment in these programs, not only financially but also through policy and educational support, as these will be essential to their ongoing success.

### Supplementary Information

Supplementary Material 1.

## Data Availability

The datasets generated and analysed during the current study are not publicly available as outlined in our ethics application but are available from the corresponding author on reasonable request and/or upon the approval of local implementation partners. The de-identified is held in RDN secure files as outlined in ethics proposal.
