# Supplementary material for: Factors associated with satisfaction of the australian rural resident medical officer cadetship program: results from a cross-sectional study
Source: BMC Med Educ. 2024 Jul 29;24:816. doi: 10.1186/s12909-024-05737-z (PMC11288089; doi:10.1186/s12909-024-05737-z)
Supplement: Supplementary file 1 — Supplementary Material 1. [file 12909_2024_5737_MOESM1_ESM.docx]

| 1. Name | 31. if you had not received the cadetship, would you still have applied for a rural internship?  a. Yes  b. No |
| --- | --- |
| 1. Gender    1. Female    2. Male    3. Other | 32. please give reason for your answer. |
| 1. Name of University | 33. Did you remain in the same area after completing your Return of Service? |
| 1. In which location did you predominantly grow up?    1. Rural    2. Regional    3. Remote    4. Metropolitan    5. Overseas | 34. If yes, for how long? (years, months) |
| 1. Where did you do most of your primary schooling    1. Rural    2. Regional    3. Remote    4. Metropolitan    5. Overseas | 35. If no, why not? |
| 1. Where did you do most of your secondary schooling?    1. Rural    2. Regional    3. Remote    4. Metropolitan    5. Overseas | 36. Did your time in a Regional Hospital influence your career decisions?  a. Yes  b. No |
| 1. Have you completed you Return of Service Obligation? If so, year of completion. | 37. Please give reasons for your answer. |
| 1. If no, reason for withdrawal | 38. How important was the Cadetship and period of rural service in influencing your decision to practice rurally? |
| 1. What was the location for your Return of Service? | 39. Please give reason for your answer. |
| 1. Did you obtain any other scholarships whilst studying, for example a John Flynn or Bush Bursary scholarship. | 40. Would you recommend the cadetship to current medical students? |
| 1. If yes, please provide detail: | 41. Please give reasons for your answer |
| 1. Did you undertake rural placement through your university? | 42. Are you currently on a vocational training program?  a. Yes  b. No |
| 1. Placement 1 – location | 43. If so, which one? |
| 1. Placement 1 – length of placement (months) | 44. Comment |
| 1. Placement 2 – location | 45. Where did you undertake training? |
| 1. Placement 2 – length of placement (months) | 46. Have you completed a vocational training program?  a. Yes  b. No |
| 1. Placement 3 – location | 47. If so, which one? |
| 1. Placement 3 – length of placement (months) | 48. Did you have the option to complete any training in a rural area? |
| 1. Placement 4 – location | 49. If yes, did you complete all of it rurally?  a. Yes  b. No |
| 1. Placement 4 – length of placement (months) | 50. If no, if there was an option to complete training rurally, would you have?  a. Yes  b. No |
| 1. Placement 5 – location | 51. What is your current vocation? |
| 1. Placement 5 – length of placement (months) | 52. Which of the following described the location in which you are predominantly working?   - 1. Rural   2. Regional   3. Remote   4. Metropolitan   5. Overseas |
| 1. Did you attend a Rural Clinical School    1. Yes    2. No | 53. If metropolitan or overseas, what made you choose not to work in rural or remote Australia? |
| 1. If so, where | 54. If metropolitan or overseas, do you have any links to rural medicine e.g. locum, outreach or telehealth?  a. Yes  b. No |
| 1. And for how long? (months) | 55. Please describe |
| 1. Were you a member of your Rural Health Club?    1. Yes    2. No | 56. If rural, regional, or remote, what were the main influenced in making you choose to pursue a rural career? |
| 1. What were the main influences in making you apply for cadetship> (select multiple if applicable)    1. Financial support    2. Networking opportunities    3. Support offered by RDN    4. Desire to do PGY rurally    5. Long-term rural intent    6. Improve chance of specialty position    7. Out of comfort zone    8. Other: please comment | 57. What is your intention to practice rurally in the future?  a. Very unlikely  b. Unlikely  c. Neutral  d. Likely  e. Very likely |
| 1. What were the main advantages of holding a Cadetship? | 58. What were the main influenced in your continued interest in rural health? |
| 1. What were the disadvantages? | 59. Are there elements of the cadetship that could be improved upon? |
| 1. How would you rate your overall experience of the Cadetship?    1. Very poor    2. Poor    3. Average    4. Good    5. Excellent | End of survey |
